# Association of exposure to salinity in groundwater with chronic kidney disease among diabetic population in Bangladesh

**DOI:** 10.1371/journal.pone.0284126

**Published:** 2023-04-11

**Authors:** Rajib Mondal, Palash Chandra Banik, Mithila Faruque, Saidur Rahman Mashreky, Liaquat Ali

**Affiliations:** 1 Department of Public Health, Hamdard University Bangladesh, Munshiganj, Bangladesh; 2 Department of Noncommunicable Diseases, Bangladesh University of Health Sciences (BUHS), Dhaka, Bangladesh; 3 Department of Biochemistry and Cell Biology, Bangladesh University of Health Sciences (BUHS), Dhaka, Bangladesh; Delta State University, NIGERIA

## Abstract

Globally, chronic kidney disease (CKD) is one of the major public health concerns. CKD and renal failure are reported to be high in the areas with higher salinity, however, the association is still unclear. We aimed at assessing the association of degree of groundwater salinity with CKD among diabetic populations of two selected areas in Bangladesh. This cross-sectional analytic study was carried out among 356 diabetic patients aged 40–60 years in high groundwater salinity exposed Pirojpur (n = 151) and non-exposed Dinajpur (n = 205), the southern and northern districts of Bangladesh, respectively. The primary outcome was the presence of CKD (via estimated glomerular filtration rate <60 ml/min) using Modification of Diet in Renal Disease equation. Binary logistic regression analyses were done. In non-exposed (mean age 51.2±6.9 years) and exposed (mean age 50.8±6.9 years) respondents, men (57.6%) and women (62.9%) were predominant, respectively. The proportion of patients with CKD was found to be higher in the exposed group than that of the non-exposed group (33.1% vs. 26.8%; *P* 0.199). The odds (OR [95% confidence interval]; *P*) of CKD were not found to be significantly higher in high salinity exposed respondents (1.35 [0.85–2.14]; 0.199), compared to the non-exposed. However, the odds of hypertension were found to be significantly higher in high salinity exposed respondents (2.10 [1.37–3.23]; 0.001), compared to the non-exposed. And, the interaction of high salinity and hypertension showed a significant association with CKD (*P* = 0.009). In conclusion, the findings suggest that groundwater salinity may not be directly associated with CKD in southern Bangladesh, however, it may have an indirect association with the disorder through the association of hypertension with groundwater salinity. Further large scaled studies are required to answer the research hypothesis more clearly.

## Introduction

Chronic kidney disease (CKD) is one of the major public health concerns [1]. Globally, 8–16% of the people are suffering from CKD [2, 3]. Over 2 million people receive treatment with dialysis or a kidney transplant to stay alive, yet this number may only represent 10% of people who actually need treatment to live [4]. The burden of CKD is also noticeable among the South Asian populations, with an estimated rate of 10.6–23.3% [5]. Particularly in Bangladesh, the prevalence of CKD has been found notably higher (19–26%) than the global context [6, 7]. However, diabetes mellitus and hypertension are the most common cause of CKD [8, 9], and can easily and quickly progress to end-stage kidney disease [9].

High salt or sodium intake has been found to have a potential association with an increased risk of kidney diseases [10, 11]. Clinical evidence also revealed that excessive salt intake accelerates the adverse renal function in hypertensive patients [12, 13]. In other ways, statistics report that salinity of drinking groundwater in coastal areas may cause hypertension [14–17]. However, there is a dearth of clear knowledge whether groundwater salinity is associated with CKD. Palestinian researchers sought for the association of renal failure prevalence among general population with salinity of drinking water along with several hydro-chemical compounds in the southern part in Gaza Strip, and they didn’t find significant association with sodium [18]. Similarly, there was no significant association of CKD prevalence with sodium in drinking water among the general population of North-Central Province of Sri Lanka, which experiences CKD epidemic [19]. However, there was a significant association with fluoride level [18] and fluoride-sodium combination [19].

In Bangladesh, nearly one-third of its districts (19 out of 64) located in the southern coastal areas have been marked for the most vulnerable areas due to salinization which occupy a large number of its total population [20]. Moreover, nearly 9% of this coastal population is affected by diabetes [21]. Conceptually, this large number of diabetic populations is supposed to be more vulnerable for CKD due to salinity issue. However, no study sought for this highly important context exploring the association of groundwater salinity with CKD, to the best of available online published data. The objective of this study was to assess the association of degree of groundwater salinity with CKD among diabetic population of two selected areas in Bangladesh.

## Materials and methods

### Study design, setting and population

This cross-sectional analytic study was carried out in 2017 in Alhaz Asmat Ali Khan Diabetic Hospital, Pirojpur, and Dinajpur Diabetic Hospital, Dinajpur districts. Both hospitals are affiliated with the Diabetic Association of Bangladesh (BADAS). A total of 356 local and registered diabetic patients (151 from Pirojpur and 205 from Dinajpur) aged 40–60 years and had a history of type-2 diabetes mellitus for at least 3 years, were enrolled from the outdoors conveniently.

Pirojpur is one of the riverine districts located in the southern part of the country and around 40 km away from Mongla Sea Port, which is situated on the shore of Bay of Bengal [22, 23]. This district was selected purposively as it is one of the 19 vulnerable coastal districts of the country exposed to high salinity because of geographical location nearer to the Bay of Bengal [20]. In contrast, Dinajpur is in the northern part of the country and around 365 km away from Mongla Sea Port [22, 23], selected purposively as the high salinity non-exposed area.

### Exposure, outcomes, and other covariates

High groundwater salinity was the primary exposure, representing the respondents from Pirojpur district [20]. The primary and secondary outcomes were the presence of CKD (proportion and association between exposed and nonexposed groups) and hypertension, respectively. And, the respondent’s selected demographic (sex and age)-, behavioral risk factors (tobacco use, alcohol consumption, insufficient fruit and vegetables intake, and added salt intake)-, and metabolic risk factors (hypertension and blood sugar levels)-related information were considered as the covariates.

### Data collection instruments, tool and technique

A structured questionnaire was adopted from WHO STEPS instrument [24], that comprised of socio-demographic information (sex, age, education, occupation, and family income), behavioral risk factors (tobacco use [smoking and/or smokeless], alcohol consumption, insufficient fruit and vegetables intake, and added salt intake), metabolic risk factors (hypertension and overweight and/or obesity), biochemical factors (serum creatinine and fasting and/or random blood sugars), and anthropometric information (height and weight). The questionnaire was pretested before the final data collection.

The respondents were asked about their socio-demographic- and behavioral risk factors-related information. Fruit and vegetables (FAVs) intake was assessed in serving size (1 standard serving = 80 grams or 1 standard size cup of raw fruit and vegetables, or half cup of cooked vegetables) using the show-cards from STEPS instrument [24]. Recent (within 3 months) serum creatinine values, on spot fasting blood sugar (FBS) and/or random blood sugar (RBS) values, blood pressures, and recent heights and weights were taken from respondent’s personal diabetes record book. Body mass index (BMI) was calculated following the standard formula dividing the respondent’s weight (in kg) by the square of height (in meter) [24]. Estimated eGFR was used to assess their renal functions.

#### Defining CKD

The Modification of Diet in Renal Disease Study (MDRD) equation using respective respondent’s serum creatinine, sex, age, and race was used to calculate eGFR. And, eGFR<60 ml/min/1.73m^2^ was considered as the presence of CKD according to the National Kidney Foundation guideline [25].

#### Defining the behavioral and metabolic risk factors

Presence of behavioral and metabolic risk factors was defined following the guideline of STEPS instrument [24]. Insufficient FAVs intake and overweight and obesity were defined as following- FAVs <5 servings/day and BMI ≥25.0, respectively. Hypertension was defined when systolic blood pressure (SBP) was ≥140 mm and/or diastolic blood pressure (DBP) was ≥90 mm of Hg or a history of previously diagnosed hypertension.

### Statistical analysis

SPSS software (IBM Corp. Released 2013. IBM SPSS Statistics for Windows, Version 21.0. Armonk, NY: IBM Corp.) was used for data processing and statistical analysis. Descriptive statistics were done for each variable. Inferential statistics such as Chi-square or Fisher’s Exact tests and Independent Sample *t*-tests were used to see the significant differences between high salinity non-exposed and exposed groups with socio-demographic, behavioral, and metabolic risk factors. Univariable binary logistic regression analysis was done on each variable (i.e., high salinity exposure and other covariates) with CKD, individually, to compute a crude odds ratio (with 95% confidence intervals [CI]) and also to assess the associations. These covariates (sex, age, tobacco use, insufficient fruit and vegetables intake, added salt intake, hypertension, and fasting and random blood sugar) are the non-confounding covariates in our study, as per the plausible causal pathway of high salinity exposure and CKD. The covariates which showed a significant (*P*<0.05) or near significant (*P*<0.1) association with CKD (keeping sex and age common as biological factors), were used in the multivariable binary logistic regression model to explain their individual interaction terms with high salinity exposure to assess their joint effects on CKD.

### Ethical considerations

This study was conducted in accordance with the Declaration of Helsinki, and the protocol was approved by the Ethical Review Committee (ERC) of Bangladesh University of Health Sciences (Identification no. BUHS/BIO/EA/17/82). Informed consents (both verbal and written) were taken from each respondent.

## Results

### Population characteristics

Men (57.6%) and women (62.9%) respondents were predominant in non-exposed and exposed groups, respectively (*P*<0.001). The mean±SD ages of the respondents of both groups were similar (51.2±6.9 and 50.8±6.9 in non-exposed and exposed groups, respectively), and the majority of them were 50 years and older. Most of them completed primary school level and were housewives in both groups, the details in the **Table 1**.

**Table 1:** Socio-demographic characteristics of the high groundwater salinity non-exposed (*n* = 205) and exposed (*n* = 151) diabetic populations in Bangladesh (total *n* = 356).

Variables		Non-exposed, *n* (%)	Exposed, *n* (%)	χ2	*P*
**Sex**					
	Men	118 (57.6)	56 (37.1)	14.59	<0.001
	Women	87 (42.4)	95 (62.9)		
**Age**, *in years*					
	Below 50	73 (35.6)	54 (35.8)	0.01	0.976
	50 and above	132 (64.4)	97 (64.2)		
**Educational status**					
	No education	25 (12.2)	20 (13.2)		
	Up to primary	81 (39.5)	64 (42.4)	0.76	0.855
	Up to secondary	42 (20.5)	31 (20.5)		
	Higher secondary and above	57 (27.8)	36 (23.8)		
**Occupation**					
	Employed	41 (20.0)	26 (17.2)		
	Business	53 (25.9)	20 (13.2)	11.66	0.009
	Housewife	82 (40.0)	84 (55.6)		
	Others	29 (14.1)	21 (13.9)		
**Monthly family income**, *in BDT*					
	Below 30,000	83 (40.5)	66 (43.7)	0.37	0.543
	30,000 and above	122 (59.5)	85 (56.3)		

BDT = Bangladeshi Taka

### Behavioral and metabolic risk factors

In non-exposed and exposed groups, there was no significant difference of tobacco use (28.3% and 31.1%, respectively; *P* = 0.420), alcohol consumption (5.4% and 2.6%, respectively; *P* = 0.288), overweight and obesity (*P* = 0.106) and fasting blood sugar level (*P* = 0.567). However, the exposed respondents were significantly higher (*P<*0.05) with taking insufficient FAVs (93.4% vs. 81.5%), using added salt during meals (68.9% vs. 28.3%), hypertension (63.6% vs. 45.4%) and random blood sugar level than their counterpart (**Table 2**).

**Table 2:** Behavioral and metabolic risk factors of the high groundwater salinity non-exposed (*n* = 205) and exposed (*n* = 151) diabetic populations in Bangladesh (total *n* = 356).

Variables		Non-exposed group	Exposed group		*P*
		Number (%)		χ2 value	
**Current tobacco use**					
	Yes	58 (28.3)	47 (31.1)	0.336	0.562
	No	147 (71.7)	104 (68.9)		
**Alcohol consumption**					
	Yes	11 (5.4)	4 (2.6)	1.59	0.288
	No	194 (94.6)	147 (97.4)		
**Insufficient fruit and vegetables intake** *(<5servings/day)*					
	Yes	167 (81.5)	141 (93.4)	10.59	0.001
	No	38 (18.5)	10 (6.6)		
**Added salt use during meal**					
	Yes	58 (28.3)	104 (68.9)	57.75	0.001
	No	147 (71.7)	47 (31.1)		
**Hypertension**					
	Yes	93 (45.4)	96 (63.6)	11.58	0.001
	No	112 (54.6)	55 (36.4)		
**Overweight and obesity** (*Body mass index ≥25*.*0*)					
	Yes	78 (38.0)	45 (29.8)	2.62	0.106
	No	127 (62.0)	106 (70.2)		
		**Mean±standard deviation**		***t* value**	
Fruit and vegetables intake servings/day		3.8±1.0	3.3±1.0	4.35	<0.001
Systolic blood pressure		126±11	130±14	2.54	0.011
Diastolic blood pressure		82±7	84±7	2.52	0.012
Body mass index		24.2±3.4	23.9±4.9	0.55	0.586
Fasting blood sugar (*n* = 145)		8.2±2.2	8.5±3.4	-0.575	0.567
Random blood sugar (*n* = 223)		11.5±4.1	13.8±4.6	-3.708	<0.001

Chi-square or Fisher’s Exact test (for categorical data) and Independent Sample t-test (for continuous data) were done.

Five servings = 400 gm.

### Inferential analyses: Relationship between high salinity and CKD

There was no significant difference of mean±SD eGFR between non-exposed and exposed groups (67.2±17.0 and 65.3±15.1, respectively; *P* = 0.290). The proportion of CKD was found to be higher in the exposed group than that of the non-exposed group (33.1% vs. 26.8%; *P* = 0.199), although the difference was not significant (not shown in table).

### Associations of high salinity and other covariates with CKD

**Table 3** shows the unadjusted and adjusted (along with interactions with potential covariates) associations of high salinity with CKD. When compared with non-exposed respondents, the crude odds (95% CI; *P*) of CKD was found to be 1.35 (0.85–2.14; 0.199) times higher in exposed respondents, reflecting no significantly positive association. The crude odds (95% CI; *P*) of CKD were found to be significantly 2.21 (1.38–3.54; 0.001) times higher in women, which was 1.05 (1.01–1.08; 0.009), 2.87 (1.77–4.65; <0.001), and 2.50 (1.54–4.04; <0.001) times higher with age in year, current tobacco use, and hypertension, respectively **(S1 Table)**. The associations were not potentially significant with other covariates. However, the interaction of high salinity and hypertension showed a significant association with CKD (**Table 3**).

**Table 3:** Crude and adjusted odds ratios of CKD by high groundwater salinity and interactions with other potential covariates (total *n* = 356; non-exposed 205; and exposed 151).

Crude and unadjusted models	Odds ratio	95% Confidence interval	*P*	Interaction *P*
Model 1 (high salinity)	1.35	0.85–2.14	0.199	-
Model 2	1.60	0.75–3.38	0.222	0.295
Model 3	6.41	0.17–243.74	0.317	0.404
Model 4	1.64	0.91–2.98	0.101	0.250
Model 5	2.68	1.23–5.85	**0.013**	**0.009**

Model 1 = crude (unadjusted) association of high groundwater salinity with chronic kidney disease

Model 2 = Model 1 + adjusted for sex

Model 3 = Model 1 + adjusted for age

Model 4 = Model 1 + adjusted for current tobacco use

Model 5 = Model 1 + adjusted for hypertension

### Associations of high salinity and other covariates with hypertension

On the other hand, the crude odds (95% CI; *P*) of hypertension were found to be significantly 2.10 (1.37–3.23; 0.001) times higher in exposed respondents compared to the non-exposed respondents, reflecting a significantly positive association of high groundwater salinity with hypertension (Table 4). The crude odds (95% CI; *P*) of hypertension were found to be significantly 1.04 (1.01–1.07; 0.013), 1.86 (1.17–2.98; 0.009), and 2.08 (1.11–3.89; 0.022) times higher with age in year, current tobacco use, and insufficient fruit and vegetables intake, respectively (S1 Table). And, the interaction of high salinity and current tobacco use showed a significant association with CKD (Table 4).

**Table 4:** Crude and adjusted odds ratios of hypertension by high groundwater salinity and interactions with other potential covariates (total *n* = 356; non-exposed 205; and exposed 151).

Crude and unadjusted models	Odds ratio	95% Confidence interval	*P*	Interaction *P*
Model 1 (high salinity)	2.10	1.37–3.23	0.001	-
Model 2	1.43	0.75–2.71	0.277	0.110
Model 3	3.60	0.14–93.40	0.440	0.756
Model 4	3.01	1.80–5.11	**<0.001**	**0.009**
Model 5	1.14	0.27–4.76	0.854	0.429

Model 1 = crude (unadjusted) association of high groundwater salinity with hypertension

Model 2 = Model 1 + adjusted for sex

Model 3 = Model 1 + adjusted for age

Model 4 = Model 1 + adjusted for current tobacco use

Model 5 = Model 1 + adjusted for Insufficient fruit and vegetables intake

## Discussion

The current study is a paradigm in Bangladesh as well as a global perspective that sought for the first time the association of groundwater salinity with CKD among diabetic population. Climate change-induced global warming is causing sea-level rising that leads to intrusion of saline water into the coastal regions, consequently the high salinity in drinking water may threaten human health. This present study explored how groundwater salinity is associated with CKD among the diabetic subjects of the selected southern coastal areas in Bangladesh, who are considered as the most vulnerable population for developing CKD.

In this study, we found a higher proportion of CKD among the population of high groundwater salinity exposed Pirojpur district than that of the counter district, although the difference was nonsignificant. And, there was no significantly positive association of high groundwater salinity with CKD among them, likewise the nearly relevant studies in Palestine [18] and Sri Lanka [19]. However, we found the interaction of groundwater salinity and hypertension to be significantly associated with CKD. And, there was a significantly positive association of salinity with hypertension found in this study. This finding has been found to be consistent with the other domestic studies that found higher prevalence of hypertension among the population in high salinity exposed coastal areas [14–17]. The nature of these findings in our study and the above-mentioned studies suggests that although the high groundwater salinity may not be directly associated with CKD among the population, it may increase the risk of CKD among them through developing hypertension. Meanwhile, the significantly positive association of the interaction of groundwater salinity and tobacco use with hypertension (with relatively higher level of significance than that of association between salinity and hypertension) that we observed also indicates that tobacco use may increase the risk of developing hypertension among the high groundwater salinity-exposed population.

There might be a few potential factors that may interfere with the association of high groundwater salinity with CKD in this study. Sex difference might have an impact, as women were with higher CKD (**Table 3**). The considered high salinity exposed area in this study (Pirojpur) might not have enough groundwater salinity concentration to cause CKD. In addition, the prevalence of CKD in non-exposed group of this study has been found remarkably higher than the below 65-years old diabetic population in other studies from several countries, including neighboring country India [26–30]. This suggests that the non-exposed diabetic population of this study may also be exposed to other factor(s) rather than the salinity issue, that may contribute to the CKD vulnerability. Report also suggests that CKD is epidemic in low humidity, drier, heat-stressed and low rainfall geographical areas [31]. The recent CKD epidemic areas around the world are- the North-Central Province of Sri Lanka, Central America, Andhra Pradesh and Maharashtra of India, North-Eastern (Isan) region of Thailand, Tierra Blanca of Mexico, and few areas of Northern Africa and Middle East, whereas all of the regions are characterized by dry zone, hottest, low humidity and low rainfall areas [31]. In the same way, the non-exposed area of this study is also characterized by lowest humidity and lowest annual average rainfall along with higher temperature compared to other geographical distributions of the country [32]. This climatic issue might be an important reason for noticeable CKD prevalence in the non-exposed area. In addition, although the added salt intake behavior during meals has been found significantly higher among the exposed population, however the proportion of this behavior among the exposed population is consistent with the prevalence found from other domestic studies [33–36]. Therefore, the inconsistent proportion of this behavior in the non-exposed population might be due to the underreporting or past use.

There were a few limitations in this study that might affect the results. It was inconvenient to measure the actual concentration of groundwater salinity of the study areas, as this was a self-funded student work. It was also not possible to eliminate all of the potential interfering factors mainly- amount of dietary salt intake, issues of other hydro-chemical compounds and the climate of the study areas. However, this preliminary baseline study revealed a comparatively higher proportion of CKD in high groundwater salinity exposed areas than the counterpart, which can be considered as the driving fuel for the researchers for further epidemiologic studies with the same research hypothesis. All of the considered confounding factors in this study were adjusted in logistic regression. Positive association of groundwater salinity with hypertension can be considered as an indirect association of salinity with CKD. This study showed the avenue for further epidemiologic studies addressing the potential factors.

## Conclusion

The data suggest that groundwater salinity may not be directly associated with CKD in southern Bangladesh, however it may have an indirect association with the disorder through the association of hypertension with groundwater salinity in the area. Further large scaled studies with measuring the groundwater salinity concentration are required to answer this research hypothesis more clearly.

## Supporting information

S1 File (SAV)

S1 Table Crude associations of CKD and hypertension with covariates (total *n* = 356; non-exposed 205; and exposed 151). (DOCX)
