# Supplementary material for: Association of exposure to salinity in groundwater with chronic kidney disease among diabetic population in Bangladesh
Source: PLoS One. 2023 Apr 11;18(4):e0284126. doi: 10.1371/journal.pone.0284126 (PMC10089349; doi:10.1371/journal.pone.0284126)
Supplement: S1 Table — (DOCX) [file pone.0284126.s002.docx]

**S1 Table: Crude associations of CKD and hypertension with covariates (total *n*=356; non-exposed 205; and exposed 151)**

| **Independent variables** | | **CKD** | | | **Hypertension** | | |
| --- | --- | --- | --- | --- | --- | --- | --- |
|  |  | **OR** | **95% CI** | ***P*** | **OR** | **95% CI** | ***P*** |
| **Sex** | |  |  |  |  |  |  |
|  | Men (ref) |  |  |  |  |  |  |
|  | Women | 2.21 | 1.38-3.54 | **0.001** | 1.17 | 0.77-1.77 | 0.473 |
| **Age** (years) | | 1.05 | 1.01-1.08 | **0.009** | 1.04 | 1.01-1.07 | **0.013** |
| **Current tobacco use** | |  |  |  |  |  |  |
|  | No (ref) |  |  |  |  |  |  |
|  | Yes | 2.87 | 1.77-4.65 | **<0.001** | 1.86 | 1.17-2.98 | **0.009** |
| **Insufficient fruit and vegetables intake** | | | |  |  |  |  |
|  | No (ref) |  |  |  |  |  |  |
|  | Yes | 1.02 | 0.52-1.99 | 0.957 | 2.08 | 1.11-3.89 | **0.022** |
| **Added salt intake** | |  |  |  |  |  |  |
|  | No (ref) |  |  |  |  |  |  |
|  | Yes | 1.19 | 0.75-1.88 | 0.453 | 0.96 | 0.63-1.45 | 0.830 |
| **Presence of hypertension** | |  |  |  |  |  |  |
|  | No (ref) |  |  |  |  |  |  |
|  | Yes | 2.50 | 1.54-4.04 | **<0.001** | - | - | - |
| **Fasting blood sugar** | | 1.08 | 0.95-1.22 | 0.253 | 1.01 | 0.90-1.15 | 0.764 |
| **Random blood sugar** | | 1.00 | 0.93-1.06 | 0.888 | 0.99 | 0.93-1.05 | 0.652 |

CKD= chronic kidney disease; OR=odds ratio; CI= confidence interval; ref= reference
